# News and Events

**Published:** 2008

**Authors:** T. Ganesh

**Affiliations:** Department of Radiation Oncology, King Fahad Specialist Hospital, Dammam, Saudi Arabia. E-mail: sptgnadar@yahoo.com

## United Kingdom: Report on incidents involving excessive dose to patients

United Kingdom has released a compilation of incidents in which a number of patients have received doses of radiation much greater than intended. The document (*http://www.healthcarecommission.org.uk/_db/_documents/IRMER_14month_report.pdf*) gives an insight into the type of incidents that occurred in the fields of diagnostic radiology, nuclear medicine, and radiotherapy during the period from November 2006 to December 2007.

## Recent Publications of Interest from International Atomic Energy Agency (IAEA)

### Training Materials on Radiological Protection of Patients: Cardiology

In its continued efforts to improve the radiological protection of patients, IAEA has recently added training material on Cardiology on its website *http://rpop.iaea.org/RPOP/RPoP/Content/AdditionalResources/Training/1_TrainingMaterial/index.htm* The site has excellent teaching and training materials on Diagnostic and Interventional Radiology, Radiotherapy, Nuclear Medicine, and Prevention of Accidental Exposure in Radiotherapy.

### Imaging in Radiotherapy

The May 2008 issue of the IAEA-Secondary Standards Dosimetry Laboratories (SSDL) Newsletter (No. 55) has an exhaustive review article on Imaging in Radiotherapy in the form of Report of a Consultants′ Meeting (Vienna, October 15-19, 2007). The entire newsletter issue can be downloaded using the link *http://www-pub.iaea.org/MTCD/publications/PDF/Newsletters/SSDL-NL-55.pdf*.

### ‘You can't wait forever!’

“Most countries need to take this decision. You can't wait forever. We don't say nuclear power is the only solution, but it is one solution”, says the project manager of the world's largest nuclear reactor about the Finland's decision to take nuclear path for tackling energy crisis. Olkiluoto 3, or simply OL3, under construction at Eurajoki in the western coast of Finland will ultimately generate 1600 MW of electricity with an Indian-designed turbine hall.

Finns have taken the extraordinary step of turning the reactor into a tourist attraction site drawing more than 20,000 visitors per annum including questioning politicians from disparate countries and curious kindergarten children. The aim is to pre-empt all apprehensions by way of giving information — lots of it, simply and clearly put!

The Finnish parliament voted to build OL3 in 2002, in a decision seen as revolutionary in a Europe that had built no new nuclear plants since the 1986 Chernobyl accident and where opposition to nuclear power has traditionally been fierce. But OL3′s strict counter-terrorism safety features — post-9/11, a plane could be crashed into it but the reactor will not leak — and calm agreement on nuclear waste disposal appear to be winning European hearts and minds. The reactor OL3 will be ready in 2011.

From the Times of India dated June 12, 2008

### China Earthquake Buried Many Radiation Sources

The May 12, 2008, earthquake in the Sichuan province of China that measured 8.0 on Richter scale and killed nearly 75,000 people had yet another casualty in the form of buried radioactive sources. According to state-run Xinhua news agency, about 50 sources have been buried in the debris of earthquake and nearly 35 of them have been ‘secured’ back. The news service did not elaborate on any potential threat to the public and did not provide details on what the radioactive materials were or where exactly they were found. It said only that “nuclear facilities and radioactive sources for civilian purposes … have been confirmed safe and controllable.” A French nuclear expert said the radioactive sources likely came from materials used in hospitals, factories, or in research—not for weapons.

From National Geographic News dated May 30, 2008

### MRI technique to catch cancer early

Researchers at the Kevin Brindle of the University of Cambridge have developed a new imaging technique that can help detecting cancer early. The noninvasive method uses magnetic resonance imaging to measure changes in pH — or acidity — in tissue that is often the hallmark of cancer and other conditions such as heart disease and strokes.

Tumors are more acidic and measuring the pH levels in tissues can help to differentiate between cancerous tissue and normal tissue. The researchers injected mice with a tagged form of bicarbonate — an alkali more commonly seen in baking soda — that occurs naturally in the body and balances acidity. They used MRI to see how much of the tagged bicarbonate was converted into carbon dioxide within the tumor. The researchers measured pH levels using an emerging technique called dynamic nuclear polarization that boosts MRI sensitivity more than 10,000 times.

The method involves cooling down molecules to near absolute zero and then warming them up quickly — a process that keeps them polarized and easier to detect as an image.

From the Times of India dated May 30, 2008

## New Publication from NCRP: NCRP Report No. 157, Radiation Protection in Educational Institutions

The purpose of NCRP Report No. 157, Radiation Protection in Educational Institutions, is to provide guidance for the safe use of ionizing- and nonionizing-radiationsources in educational institutions, including both teaching and research activities. To take advantage of the benefits of using radiation sources, it is necessary to provide radiation safety controls commensurate with the potential hazard. Since the sources of radiation used in many educational institutions usually produce only low radiation levels, the potential hazard to faculty, staff, and students is usually correspondingly low when simple basic precautions are followed.

This report provides detailed information to the administrators on determining whether a radiation safety program is necessary, and to assist the Radiation Safety Officer (RSO) in assuming the responsibility for the radiation safety program.

## National Academy of Sciences/National Research Council (NAS/NRC) publishes report on Radiation Source Use and Replacement

The 9/11 terrorist attacks on WTC Towers using passenger airplanes have forced the administrators and security experts to seriously consider the possibility that other technologies, which were designed and are used solely for the benefit of society, could be used in a destructive manner. Radiological hazard, resulting from inappropriate usage of radiation sources and/or radiation-generating machines, ranks high among the several other concerns.

Concern about the safety and security of high-intensity radiation sources grew particularly amid fears that terrorists might use radiation sources to make a radiological dispersal device or ‘dirty bomb’. As a part of addressing such growing concerns about illegal use of radiation sources, the U.S. Congress asked the National Research Council to review the civilian uses of radionuclide radiation sources and potential replacements for sources that pose a high risk to public health or safety in the event of an accident or attack.

Of the several findings and recommendations thereupon, the council's observations on cesium sources in general and cesium blood irradiators in particular are worth a closer look in the Indian context. Pointing to its dispersibility, solubility, penetrating radiation, source activity, and increased presence across the country, the council has concluded that the cesium chloride sources posed unique risks. The council went on to recommend replacing these sources in US and to the extent possible globally. Further it suggested that the recommendations can be implemented by way of discontinuing licensing; incentives for decommissioning (of cesium chloride sources); prohibiting the export of cesium chloride sources to other countries. The findings and recommendations of the council can be downloaded from *http://www.nap.edu/catalog.php?record_id=11976*.

Such safety and security concerns cannot be confined to just one country only. They are equally true in all countries that have experienced terrorism in one form or the other, like India. Unfortunately, we cannot afford to take comfort under the umbrella that we have an efficient regulatory mechanism in force and be complacent. Though it is not necessary to follow the footsteps of another country, the issue is real and we do have to look into it to develop a long-term policy.

## Let us image gently!

The Alliance for Radiation Safety in Pediatric Imaging, of which American Association of Physicists in Medicine (AAPM) is a founding member, launched the ‘Image Gently’ campaign on January 22, 2008. The Alliance's goal is to change practice: to raise awareness of the opportunities to lower radiation dose in the imaging of children. The Alliance's strategy to meet the goal is straightforward information provided to every member of the care team.

We, as health care professionals involved in the field, can contribute significantly by spearheading the campaign in our country and reduce radiation doses to pediatric patients by reassessing CT protocols for children followed in our workplaces. Please visit the website *www.imagegently.org* for further information and downloading of CT protocols and other valuable information.

## U.S. Food and Drug Administration (FDA) Preliminary Public Health Notification: Possible Malfunction of Electronic Medical Devices Caused by Computed Tomography (CT) Scanning

On July 14, 2008, FDA has alerted all health care professionals to the possibility that the x-rays used during CT examinations may cause some implanted and external electronic medical devices to malfunction, and to provide recommendations to reduce the potential risk. FDA has received a small number of reports of adverse events in which CT scans may have interfered with electronic medical devices, including pacemakers, defibrillators, neurostimulators, and implanted or externally worn drug infusion pumps.

The adverse events reported to the FDA include unintended shocks from neurostimulators, malfunctions of insulin infusion pumps, transient changes in pacemaker output pulse rate. All these adverse events are presumed to have been caused by x-rays from CT scan. FDA has come out with several important recommendations, which can be viewed at *http://www.fda.gov/cdrh/safety/071408-ctscanning.html*.

### National

1. **International Conference on Medical Physics — 2008 and 29^th^ Annual Conference of Association of Medical Physicists of India**
  November 26-29, 2008, Mumbai, India
  Abstract Submission Deadline: July 31, 2008
  For more details, log onto: www.icmp2008.com
2. **30^th^ Annual Conference of the Association of Radiation Oncologists of India**
  November 27-30, 2008, Mumbai, India
  Abstract Submission Deadline: August 30, 2008
  For more details, log onto: www.aroicon2008.com
3. **9^th^ Asia Oceania Congress of Nuclear Medicine and Biology**
  October 31-Nov 4, 2008, New Delhi, India
  For more details, log onto: http://www.aofnmb2008.in/
  E-mail: admin@aofnmb2008.in

### International

1. **International Conference on Advances in Radiation Oncology (ICARO)**
  Meeting Dates: April 27-29, 2009, Vienna, Austria
  Abstract Submission Deadline: October 15, 2008
  For more details, log onto: http://www-pub.iaea.org/MTCD/Meetings/Announcements.asp?ConfiD=35265
  E-mail: ICARO@iaea.org
2. **Visions and Perspectives in Image-Guided Radiation Oncology — A Meeting for Physicians, Physicists, and Computer Scientists**
  Meeting Dates: September 24-26, 2008, Vienna, Austria
  For more details, log onto: http://www.meduniwien.ac.at/igrtvienna08/
3. **Joint ICTP-IAEA Activity on Imaging in Advanced Radiotherapy Techniques**
  Meeting Dates: October 20-24, 2008, Trieste, Italy
  For more details, log onto: http://mpekrak08.ftj.agh.edu.pl/

